# Chimeric Antigen Receptor-T Cells: A Pharmaceutical Scope

**DOI:** 10.3389/fphar.2021.720692

**Published:** 2021-08-20

**Authors:** Alejandrina Hernández-López, Mario A. Téllez-González, Paul Mondragón-Terán, Angélica Meneses-Acosta

**Affiliations:** ^1^ Laboratorio 7 Biotecnología Farmacéutica, Facultad de Farmacia, Universidad Autónoma Del Estado de Morelos, UAEM, Cuernavaca, Mexico; ^2^ Coordinación de Investigación, Centro Médico Nacional “20 de Noviembre” ISSSTE, Mexico city, Mexico

**Keywords:** CAR-T cells therapy, cell and gene therapy, advanced cell therapy, cell manufacturing process, immunotherapy

## Abstract

*Cancer* is among the leading causes of death worldwide. Therefore, improving cancer therapeutic strategies using novel alternatives is a top priority on the contemporary scientific agenda. An example of such strategies is immunotherapy, which is based on teaching the immune system to recognize, attack, and kill malignant cancer cells. Several types of immunotherapies are currently used to treat cancer, including adoptive cell therapy (ACT). Chimeric Antigen Receptors therapy (CAR therapy) is a kind of ATC where autologous T cells are genetically engineered to express CARs (CAR-T cells) to specifically kill the tumor cells. CAR-T cell therapy is an opportunity to treat patients that have not responded to other first-line cancer treatments. Nowadays, this type of therapy still has many challenges to overcome to be considered as a first-line clinical treatment. This emerging technology is still classified as an advanced therapy from the pharmaceutical point of view, hence, for it to be applied it must firstly meet certain requirements demanded by the authority. For this reason, the aim of this review is to present a global vision of different immunotherapies and focus on CAR-T cell technology analyzing its elements, its history, and its challenges. Furthermore, analyzing the opportunity areas for CAR-T technology to become an affordable treatment modality taking the basic, clinical, and practical aspects into consideration.

## Introduction

*Cancer* is a major health issue worldwide, causing a serious health problem that affects costs on public health, social development and, most importantly, reducing people’s life expectancy (Bray et al., 2018; Hassanpour and Dehghani 2017). In a broader context, cancer refers to more than 277 different types of cancer diseases (Hassanpour and Dehghani 2017), which represent major challenges to find out specific diagnosis and treatment. Worldwide, the main incidence of cancer types in adults occurs in the prostate, lung, bronchus, colon, rectum, urinary bladder, breast, and thyroid (Siegel et al., 2019), meanwhile, blood cancer is the main cancer type of concern in children´s health (Kolonel and Wilkens). For years, the most effective cancer treatments have included surgery, chemotherapy, radiation therapy and bone marrow transplant (Arruebo et al., 2011), these therapies are considered as the main treatment. Unfortunately, even considering the current technological improvements on such therapies, recurrence and metastasis are still the main causes of death (Bray et al., 2018). In recent years, these common treatments have been challenged with the advent of immunotherapy.

Immunotherapies are designed to harness and manipulate part of the immune system to confer the ability to detect and specifically attack cancer cells (Waldmann 2003). Immunotherapies introduce a different perspective into cancer treatment, not only in patients with refractory tumors, but also to ensure long-lasting clinical remission in patients that were historically considered incurable (Dougan and Dranoff 2012; Guedan et al., 2019). Hence, in this paper we intend to expose the general panorama regarding immunotherapies, focusing the effort to the novel CAR-T cell therapy, its components, advantages, and limitations, summarizing the current challenges which need to be overcome and discussing the actual strategies to implement this therapy with a wider scope in benefit of a larger number of patients.

## Immunotherapy, A New Approach for Cancer Treatment

Chemotherapy, radiation, and surgery are the most conventional cancer treatments, nevertheless, they have shown low efficacy and severe adverse effects. Therefore, during the last decade, researchers have addressed the need to improve the inconveniences with conventional cancer therapy, developing new strategies to achieve complete remission of the disease using molecular strategies. Under these circumstances, immunotherapy arises as a promise for revolutionary cancer treatment, because, compared with conventional therapeutics, it leads to lower side effects and can specifically target cancer cells by regulating the immune system´s machinery (Papaioannou et al., 2016; Sang et al., 2019). Therefore, immunotherapy has become an important therapeutic alternative for patients whose immune systems are already compromised due to their advanced disease and/or failure of previous conventional therapies (Ventola, 2017; Charmsaz et al., 2019). Table 1 covers the six major kinds of available cancer immunotherapies. The most recent immunotherapy that has shown promising results is the Chimeric Antigen Receptor (CAR)-T cell therapy, which is a type of Adoptive Cell Therapy (ACT). This therapeutic strategy entails genetically engineering a patient’s T cells to express CARs that recognize, bind, attack and kill tumoral cells (Mirzaei et al., 2017).

**TABLE 1:** Six major kinds of cancer immunotherapy.

*Cancer* immunotherapy	Description	Indications	References
Checkpoint inhibitors	This treatment takes the brakes off the immune system, helping it to identify and attack cancer cells. Checkpoint inhibitor therapies have been discovered and studied for cancer treatment based on CTLA-4 and PD-1	Pleural mesothelioma, classical Hodgkin’s lymphoma, Merkel cell and cutaneous squamous cell carcinoma, head and neck cancer, triple negative breast cancer, lung, colorectal, kidney, bladder, cervical, endometrial, liver and stomach cancers, as well non-blood cancers that test positive for the biomarkers MSI-high/dMMR, tumor mutation burden high esophageal squamous cell, renal cell, urothelial carcinoma, and primary mediastinal large B cell lymphoma	Darvin et al. (2018)
			Sang et al. (2019)
			Twomey & Zhang (2021)
Cytokines	This treatment promotes the activation of several important specialized cellular functions to boost the body’s immune system	Advanced melanomas, kidney, liver, lung, head and neck, breast, ovarian, cervical, prostate, pancreatic, colorectal, bladder, gastric, brain cancers, Sarcomas, hematological malignancies	Berraondo et al. (2019)
			Parmiani et al. (2000)
			Qiu et al. (2021)
Monoclonal antibodies (mAbs)	mAbs have an unique antigen specificity that allows them to bind to specific epitopes on cancer cells and kill them by different mechanisms. For example, they can induce apoptotic cell death or the attack by NK cells due to the ADCC action	Non-Hodgkin’s and Hodgkin’s lymphoma, triple negative breast cancer, melanoma, chronic lymphocytic leukemia, non-small cell lung cancer, chronic myeloid leukemia, acute lymphoblastic leukemia, multiple myeloma, gastric, bladder, breast, colorectal cancers, hairy cell leukemia	Ventola (2017)
			Golay and Taylor (2020)
			Zahavi & Weiner (2020)
			Monajati et al. (2021)
*Cancer* vaccines	Vaccines teach and stimulate the body’s immune system to find, attack and eradicate cancer cells	Cervical, vaginal, vulvar, anal, liver, and prostate cancers	Karlitepe et al. (2015)
			Miao et al. (2021)
Oncolytic viruses	This treatment uses modified viruses, such as coxsackievirus, adenovirus, reovirus, herpes simplex virus, and measles virus to infect, replicate and kill neoplastic cells	Melanoma and lymph nodes	Anelone et al. (2020)
			Mondal et al. (2020)
			Nejad et al. (2021)
Adoptive Cell Therapy (ACT)	This treatment modifies the body’s own immune cell to become a cancer treatment drug. ACT can be deployed in different ways such as TIL, TCR, NK or **CAR-T** therapy	CAR-T therapy for B-cell precursor acute lymphoblastic leukemia, diffuse large B-cell lymphoma, relapsed or refractory follicular lymphoma, mantle cell lymphoma, relapsed or refractory large B-cell lymphomas and multiple myeloma	Maude et al. (2018)
			Guedan et al. (2019)
			Rohaan, et al. (2019)
			Schuster et al. (2019)
			Iragavarapu and Hildebrandt (2021)
			Munshi et al. (2021)

(CTLA-4, Cytotoxic T-lymphocyte-associated antigen 4; PD-1 Programmed Death 1: MSI-high, microsatellite instability-high; dMMR, mismatch repair deficient; ADCC, Antibody-dependent cellular cytotoxicity; TIL, Tumor-Infiltrating Lymphocytes; TCR, gene-modified T cells expressing novel T Cell Receptors, NK, Natural Killer cells; CAR, Chimeric Antigen Receptors).

### A Brief History of Chimeric Antigen Receptor-T Cells Development

To start with the historical review of the discovery of these new technologies, it is necessary to date the first bone marrow transplant for leukemia patients that was reported in 1957 by Thomas and colleagues (Thomas et al., 1957). A few years later, Miller J. and colleagues reported the immunological function of the timus, discovering the origin of T cells (Miller, 1961).

However, it was not until 1986, when Steven Rosenberg and colleagues reported a study about Tumor-Infiltrating Lymphocytes (TILs), this therapy consists of cellular isolation of T lymphocytes from a tumor, that is subjected to an *in vitro* culture and expansion to infiltrate them back into the tumor to be treated, hence leading to elimination or clinical resection of the tumor (Rosenberg et al., 1986; Lança and Silva-Santos, 2012; Garber 2019). This report allowed us to fix the gaze on the idea of “Patients’ own immune cells can fight their own cancer”.

Likewise, results of the successful T cell transfections of Sadelain and colleagues in 1992 and their methods publications for retrovirus-mediated gene transfer into primary T-lymphocytes in 1997, allowed genetic modifications providing the means of controlling immunity in an experimental or therapeutic setting (Sadelain and Mulligan, 1992; Sadelain, 1997). Almost at the same time, Zelig Eshhar and colleagues designed a specific activation of cytotoxic lymphocytes through chimeric single chains, using an antibody-binding domain and the γ or ζ subunits of the immunoglobulin on the T-cell receptors, developing the First Generation of CAR-T cells (Eshhar et al., 1993). Five years later, Dr. Sadelain’s group demonstrated that the integration of a co-stimulatory signal like CD28 to a CAR-T enhanced survival, proliferation and remained active, leading to the development of the Second Generation of CARs (Krause et al., 1998). The pioneering work of Dr. Carl June and his group (University of Pennsylvania) lead to the development of CAR therapy (Kalos et al., 2011), with the approach of using modified T lymphocytes that carried a CAR to target CD19^+^ leukemic B cells (Varadé et al., 2021). Such an approach was because the expression of the transmembrane glycoprotein CD19 is maintained during differentiation of B lineage at normal (14% in peripheral blood lymphocyte phenotype) (Berron-Ruiz et al., 2016) and neoplastic B cells malignancies (80% of ALL, 88% of B cell lymphomas and 100% of B cell leukemias) (Wang et al., 2012), as well as follicular dendritic cells. Nowadays, CD19 expression is widely used for the diagnosis of leukemias and lymphomas and is used as the main target for the redirection of T lymphocytes against these neoplastic associated molecules (Scheuermann and Racila 1995; Tedder, 2009; Wang et al., 2012). In 2009, Isabelle Rivière group published the manufacturing validation of biologically functional T cells targeted to CD19 antigen basing their work on promising data in the eradication of systemic B cell malignancies. This publication launched phase I clinical trials in chronic lymphocytic leukemia (CLL) and acute lymphoblastic leukemia (ALL) (Hollyman et al., 2009). CD19 expression is widely used for the diagnosis of leukemias and lymphomas and used as the main target for the redirection of T lymphocytes against these neoplastic associated molecules (Scheuermann and Racila 1995; Tedder, 2009; Wang et al., 2012) even though there may be more specific receptors for tumor cells.

The publication of the phase I clinical trial results, proving that CAR-T therapy-induced molecular remissions in adults with chemotherapy-refractory ALL (Brentjens et al., 2013; Grupp et al., 2013), was followed by a scale-up of bioprocess production. Finally, FDA approved the CD 19 CAR-T cell therapy (Tisagenlecleucel) for ALL in children and young adults (U.S. Food and Drug Administrations, 2021a). In addition, in 2021 the FDA approved the use of CAR-T cells using the B-cell maturation antigen (“BCMA” target (Idecabtagene vicleucel) in multiple myeloma (U.S. Food and Drug Administrations, 2021b).

During the first commercial lustrum of CAR immunotherapy, CAR-T therapy explored different applications in other malignancies associated with the initial biomolecules as large B-cell lymphoma (DLBCL), DLBCL arising from follicular lymphoma and high-grade B-cell lymphoma (YESCARTA U.S. Food and Drug Administrations, 2021c). So far, most studies are carried out on the discovery of other targets such as CD20, CD22, CD30 and new targets in solid tumors to increase the scope of CAR-T therapy (Elahi et al., 2018; Castella et al., 2020).

Profiting the clinical successes, the use of CAR-T cells has also been explored in a variety of cancers (Petersen and Krenciute 2019) such as diffuse large B-cell lymphoma treatments in adults and multiple myeloma (Mullard 2017; Iragavarapu and Hildebrandt 2021; Munshi, et al., 2021) in which the therapeutic products were recently approved by the Food and Drug Administration (FDA, United States). Meanwhile, the European Medicines Agency (EMA) has granted conditional approval, or their authorization is currently under review. CAR-T cells re-targeted against the ubiquitous B-cell antigen, CD19 are a remarkable innovation in the treatment of relapsed and/or refractory (R/R) B-cell acute lymphoblastic leukemia (B-ALL) (Nie et al., 2020). The success of CAR T-cells therapy in cancer is exemplified by numerous clinical trials that have shown that 70–90% complete remission (CR) can be achieved in pediatric and adult patients treated with CD19-directed CAR T-cells (Xu et al., 2019). Based on these growing CAR-T cell immunotherapy achievements, over 500 clinical trials worldwide analyzing CAR-T cells for the treatment of cancer are currently being under evaluation, and the majority are being performed in East Asia (269 trials), followed by the US (225 trials), and Europe (62 ongoing studies) (as registered at clinicaltrials.gov Q3 2020) (Albinger et al., 2021). So far, five CAR-T cell therapy agents were approved by FDA for cancer treatment: Tisagenlecleucel (Kyrmriah®) for treatment of acute lymphoblastic leukemia (ALL) (Mullard 2017), Axicabtagene ciloleucel (Yescarta™) for diffuse large B-cell lymphoma (DLBCL) and also FDA-approval for follicular lymphoma in april 2021 (Bouchkouj et al., 2019), Brexucabtagene autoleucel (Tecartus™) for treatment of mantle cell lymphoma (MCL) (Al-Juhaishi and Ahmed, 2021), Idecabtagene vicleucel (ABECMA®) for treatment of multiple myeloma (Munshi et al., 2021) and Lisocabtagene maraleucel (Breyanzi®) for diffuse large B-cell lymphoma treatment (Iragavarapu and Hildebrandt 2021) (Table 2).

**TABLE 2:** Currently approved CAR-T cells products by FDA and EMA. These are the available products up to July 2021.

Brand name	Kymriah	Yescarta	Tecartus	ABECMA	Breyanzi
Generic name	Tisagenlecleucel	Axicabtagene ciloleucel	Brexucabtagene autoleucel	Idecabtagene vicleucel	Lisocabtagene maraleucel
Indication/s	Treatment of pediatric and young adult patients (age 3–25 years) with B-cell ALL that is refractory or in second or later relapse	Treatment of adult patients with (r/r) LBCL after two or more lines of systemic therapy, PMBCL, HGBL, and DLBCL arising from FL	Treatment of adult patients with (r/r MCL)	Treatment of adult patients with R/R MM after four or more prior lines of therapy	Treatment of adult patients with r/r DLBCL after two or more lines of systemic therapy, HGBL, PMBCL, and FL grade 3B
	Adult patients with (r/r) LBCL after two or more lines of systemic therapy including DLBCL, HGBL and DLBCL arising from FL				
Target.	CD19	CD19	CD19	BCMA	CD19
Company	Novartis Pharmaceuticals Corporation	Kite Pharma/Gilead	Kite Pharma/Gilead	Bristol Myers Squibb/Bluebird bio	Juno/Bristol Myers Squibb
Price (USD)	$475,000	$373,000	$373,000	$419,500	$410,300
Approval date by FDA.	LLA; August 2017. DLBCL, HGBL and DLBCL arising from FL; april 2018	LBCL; October 2017 FL; april 2021	July 2020	March 2021	February 2021
Authorization date by EMA.	August 2018	August 2018	Conditional approval, October 2020	Recommended granting a conditional marketing authorization, June 2021	PRIME status. Marketing Authorization Application (MAA) is currently under review
Study of efficacy and safety	JULIET for DLBCL ELIANA for B-ALL	ZUMA-1	ZUMA-2	KarMMa	TRANSCEND
Clinical trial	JULIET: NCT02445248 ELIANA: NCT02435849	NCT02348216	NCT02601313	NCT03361748	NCT02631044
Enrolled participants in the clinical trial	115	307	105	149	314
Number of treated patients	79	Data not available	Data not available	Data not available	Data not available
Success rate	83% of patients achieved CR for LLA	54% of patients achieved CR.	67% of patients achieved CR.	39% of patients achieved CR.	53% of patients achieved CR.
	65% of patients achieved CR for r/r FL	82% achieved an overall response	92% achieved an overall response	81% achieved an overall response	73% achieved an overall response
Adverse effects	CRS occurred in 79% of pediatric patients, 74% in young adult patients with r/r ALL and 23% in patients with r/r DLBCL	CRS occurred in 94% of patients with LBCL including four patients who had ongoing CRS events at the time of death	CRS occurred in 91% of patients, including one fatality	CRS occurred in 85% of patients including one fatality	CRS occurred in 46% of patients including one fatality and two had ongoing CRS at the time of death
	NTX occurred in 72% of patients with r/r ALL and 58% of patients with r/r DLBCL	NTX occurred in 87% of patients with LBCL.	NTX occurred in 81% of patients	NTX of any grade occurred in 28% of patients, including one patient who had ongoing Grade 2 neurotoxicity at the time of death	NTX occurred in 35% of patients. Three patients had fatal NTX and seven had ongoing NTX at the time of death

Large B-cell lymphoma (LBCL), diffuse large B-cell lymphoma (DLBCL), High-grade B-cell lymphoma (HGBL), Follicular lymphoma (FL), Primary mediastinal large B-cell lymphoma (PMBCL), relapsed/refractory mantle cell lymphoma (r/r MCL), relapsed or refractory multiple myeloma (r/r MM), refractory follicular lymphoma (r/r FL), Cytokine release syndrome (CRS), Neurotoxicity (NTX), complete remission (CR), non-Hodgkin lymphoma (NHL), PRIority Medicines (PRIME).

### Chimeric Antigen Receptor T Cell Immunotherapy

CAR immunotherapy is a successful innovation in cancer therapies achieving impressive results in the treatment of resistant hematological malignancies highlighting its strong potential (Neelapu et al., 2017). CAR-T cell immunotherapy was developed out of the need for T cells to be able to directly recognize tumor cells without the required antigen processing or presentation by professional antigen-presenting cells (Varadé et al., 2021). This immunotherapy is based on T-cell engineering and the use of synthetic (recombinant) receptors, termed CARs, instead of the physiological (native) receptor for the antigen, the TCR (Sadelain et al., 2009). These CARs are based on a specific antibody directed to a target surface molecule and were developed with the intent of combining the tumor recognition capabilities of antibodies with the powerful antitumor effector abilities of T cells. CAR expression is most often achieved using retroviral- or lentiviral-mediated gene transfer. The T cell’s rapidly dividing nature facilitates viral integration, and its ability to establish memory serves to reinforce long-lasting transgene expression (Grosser et al., 2019). Besides, CARs constructions include all the elements necessary for intracellular signaling and activation of helper and cytotoxic T lymphocytes (Singh and McGuirk 2020).

#### Target Selection

CAR recognition of malignant cells depends on the designed chimeric recognition molecule, not on the traditional T-lymphocyte receptor (TCR) or human leukocyte antigen (HLA). This interaction between CAR and the target leads to the formation of immune synapses, where contact-dependent cytotoxicity occurs. Hence, choosing a target with high specificity and high coverage is the main objective for these therapies. Unfortunately, nowadays there is no guide with uniform criteria that shows how a target should be properly selected, however, it is important to consider that not only membrane proteins are useful, also carbohydrates and glycolipid molecules can be selected if they adapt to the clinical needs of the desired disease (Wang et al., 2017; Wei et al., 2019).

It is recommended to identify and use a target that achieves adequate coverage of tumor cells with enough specificity to prevent CAR activation in other sites that could lead to serious damage in organs. Hence, this way, it is possible to avoid the main toxic side effects in CAR-T treatment, such as cytokine release syndrome (CRS), and the “off-tumor” effect caused by damage to non-tumor cells (Liu et al., 2019).

Recently, different types of CAR have been designed to take into consideration the coverage of two selected targets and the specificity for different types of solid tumors that share proteins involved in cell malignancy. Developed strategies are known as, Pooled CAR: two CAR in the same vector; Dual CAR: cross-compliance where the first target has the signaling domain while the other one has the costimulatory domain, CAR in tandem: where two variable fraction chains are attached to the same antigen-binding domain and some others where activation or function would be inhibited by an inhibitory CAR. The previous designs will further expand CAR-T cell therapy in clinical trials (Fedorov et al., 2013; Hartmann et al., 2017). Since novel targets are currently studied, in a further section, the specific antigens are presented as a strategy to improve the CAR-T cells application (Table 3).

**TABLE 3:** Novel targets for hematologic malignancies and solid tumors.

Target	Diseases	Identifier	Status	References
	**Hematologic malignancies**			
BCMA	Multiple myeloma	NCT03502577	Recruiting, phase I	Pont et al. (2019)
		NCT04309981	Recruiting, phase I/II.	
CD38	Multiple myeloma	NCT03464916	Recruiting, phase I	Freitag, *et al.* (2020)
		NCT03767751	Recruiting, phase I/II.	Nair, *et al.* (2019)
SLAMF7	Multiple myeloma	NCT04499339	Recruiting, phase I/II.	Rodríguez-Otero et al. (2020)
CD33	Acute myeloid leukemia	NCT03971799 NCT03126864	Recruiting, phase I/II.	Freitag et al. (2020)
			Recruiting, phase I/II.	
CD123	Acute myeloid leukemia	NCT04014881	Recruiting, phase I	Hansrivijit et al. (2019)
		NCT03766126	Not recruiting, phase I	Hoffmann et al. (2018)
CD56	Multiple myeloma	NCT03473496	Recruiting	Freitag et al. (2020)
		NCT03271632	Recruiting, phase I/II.	
CD37	B- and T-cell lymphoma	NCT04136275	Recruiting, phase I/	Scarfò et al. (2018)
	**Solid tumors**			
EGFRVIII	Glioblastoma	NCT03283631	Recruiting temporally suspended, phase I	Martinez and Moon (2019)
GPC3	Hepatocellular carcinoma	NCT03146234	Not recruiting	Da Fonseca and Carrilho (2019)
GD2	Glioma, cervical cancer, sarcoma, neuroblastoma	NCT03373097	Recruiting, phase I/II.	Townsend, et al. (2018)
		NCT02761915	Recruitment completed, phase I	
HER2	CNS tumour	NCT03500991 NCT02442297	Recruiting, phase I	Mirzaei, et al. (2017)
		NCT00902044		
Mesothelin	pancreatic cancer, ovarian *Cancer*, lung *Cancer*, peritoneal carcinoma, fallopian tube cancer, mesotheliomas Pleural	NCT03054298	Recruiting, phase I	Haas et al. (2019)
		NCT03323944		
Claudin 18.2	Gastric cancer, pancreatic cancer	NCT03874897	Recruiting, phase I	Springuel et al. (2019)
		NCT03159819		
Muc1	Breast *Cancer*	NCT04020575	Recruiting, phase I	Bamdad et al. (2019)
ROR1	Breast cancer, lung cancer	NCT02706392	Recruiting, phase I	Springuel et al. (2019)

BCMA, B-cell maturation antigen; CD, cluster of differentiation; SLAMF7, SLAM Family Member 7; EGFRVIII, variant III of the epidermal growth factor receptor; GPC3, glypican 3; GD2, disialoganglioside; HER2, human epidermal growth factor receptor 2; Muc1, mucin 1; ROR1, Receptor Tyrosine kinase Like Orphan Receptor 1.

The major advantage of cell-based therapies is the variety of surface proteins that can be used as targets, which allow the specific antigen recognition in an independent manner of the major histocompatibility complex (MHC), in contrast to TCR-mediated antigen recognition (Li et al., 2018; Dwivedi et al., 2019), that deliver remarkable results in the clinic, specifically for the treatment of B cell malignancies (Grupp et al., 2013; Porter et al., 2015).

#### Chimeric Antigen Receptor Designs

**FIGURE 1:**
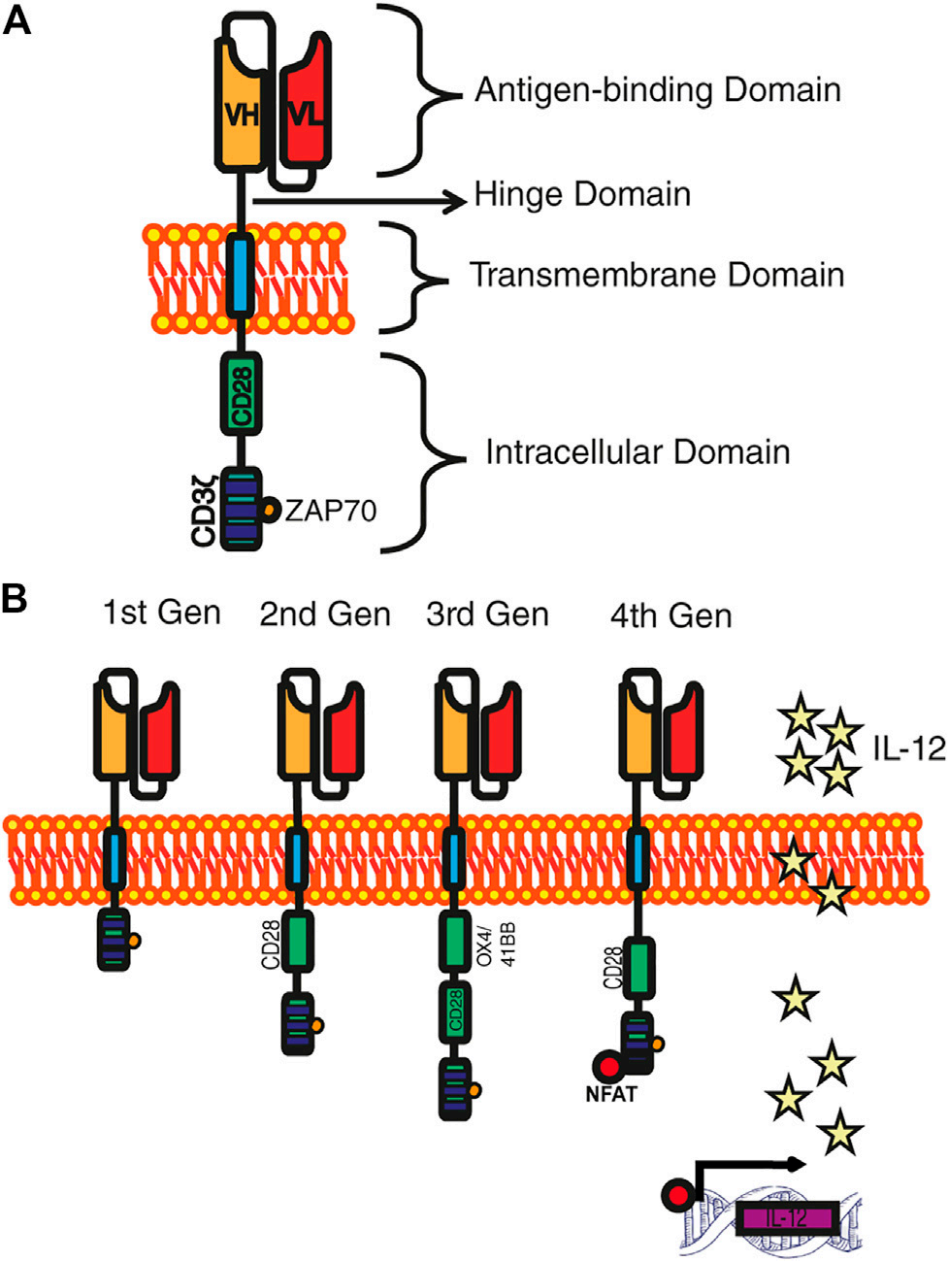
Development of CAR generations. **(A)** Archetype of a CAR. The four main domains that make up a CAR are distinguished: the recognition domain, the hinge domain, the transmembrane domain, and the intracellular domain. **(B)** CARs generations. First-generation CARs only own the CD3ζ in the intracellular domain; the second-generation CARs incorporate a costimulatory domain; the third-generation CARs include two costimulatory domains; and fourth-generation CARs are reinforced with genes that allow the expression of cytokines.

In detail, the CAR is a synthetic receptor that ligates to a surface antigen and transduces the protein target of recognition into a signaling cascade (Brown and Mackall, 2019; Qin et al., 2021). The molecular architecture of CAR consists of four main components: 1) antigen-binding (recognition) domain, which provides the exclusive recognition of the target molecule based on monoclonal antibodies with variable regions that are linked into a single-stranded fragment (scFv) (Shirasu and Kuroki, 2012); between the recognition and transmembrane domains lies a 2) hinge or spacer domain, which is a structure normally constructed by the flexible hinge sequences of CD8α, CD28 or the Fc region of immunoglobulins IgG1 or IgG4 (Lee et al., 2019; Roselli et al., 2019), this is in charge of bringing an effective immunological synapse (Fujiwara, et al., 2020). 3) A transmembrane domain, that is the anchor molecule to the cell membrane which impacts the regulation of the amount of CAR signaling into T cells via the control of the CAR surface expression level (Dwivedi et al., 2019). 4) An intracellular signaling domain, that provides the intracellular portion of the TCR invariant chain CD3ζ, which triggers the phosphorylation of the immunoreceptor tyrosine-based activation motif (ITAM´s) domains activates the signaling pathway of ZAP70 (Milone et al., 2009). However, this signal is deficient to activate by itself the resting T cell. Hence, to improve activation, proliferation, and survival the incorporation of the costimulatory domain from other receptors, such as CD28, 4-1BB (CD137), should be included to the intracellular fragment (Ramello et al., 2019) (Figure 1A).

Simple CARs classification is given by the number of signaling groups that possess in the intracellular signaling domain, being the first-generation CARs those that in their signaling domain contain CD3ζ chain as the primary transmitter of endogenous TCR signals (Ghosh et al., 2017). The first generation of chimeric receptors was primarily designed to mimic its T cell activation, a function that is sufficient to trigger cytolysis, but not enough to direct a sustained effective T cell response (Sadelain et al., 2009). To broaden T cell function, a different kind of Intracellular signaling domain was designed, mimicking a normal activation of T lymphocytes, and ensuring the presence of a co-stimulatory signal. Diverse co-stimulatory signals have been proposed such as CD28, 4-1BB and OX40, as they normally activate cell survival and proliferation pathways (i.e., PI3K–Akt–mTORC1) (Van Der Stegen et al., 2015). The second-generation of CARs have recently yielded impressive clinical results in patients with B cell malignancies, specifically in acute lymphoblastic leukemia (Brentjens et al., 2011; Maude et al., 2014). The union of two costimulatory domains in the Intracellular signaling domain is called “third-generation” CARs; this strategy increases the differentiation towards T cell effectors (Teff) and improves persistence by prolonging T-cell survival (Hombach et al., 2011). Further research has led to a novel generation of CARs named TRUCK cells (T-cells redirected for universal cytokine killing), also called “fourth generation” CARs that, in addition to costimulatory signal(s), is provided with a “nuclear factor of activated T cell-responsive expression” which can activate an innate immune response through the secretion of an element for an inducible transgenic product like IL-12 or another cytokine, (Figure 1B). This CAR-T is expected to be applied in fields of virus infections, auto-immune diseases, or metabolic disorders (Chmielewski et al., 2018; Date and Nair, 2021).

#### T-Cell Procurement

As the last element, the isolation of T cells is a crucial factor since these types of cells are needed to get a high-quality product. The requirements for CAR-T cell manufacturing process are T cells with CD3/CD4+ and CD3/CD8+ immunophenotype. For T cell harvesting the process starts from the mononuclear cell fraction notwithstanding that the final quantities of contaminating cells are dependent upon the type of blood cell isolation process, counterflow centrifugal elutriation or even the inherent properties of the patient’s blood. As a strategy for obtaining viable and high purity of T cells (CD3/CD4+ and CD3/CD8+) additional purification steps are needed; At this point, immunomagnetic isolation has been introduced to shift the density gradient separation (Fesnak and O’Doherty, 2017; Stroncek et al., 2017).

It has even been reported that the impact of CD4/CD8 T-cell selection on the starting apheresis product influences manufacturing feasibility, however, this strategy led to a direct increase in inflammatory toxicities (Shah et al., 2020a; Shah et al., 2020b). Clinical recommended applications of CAR-T-cell therapy include infusion products at a 2:1 ratio being approximately CD4^+^ CAR-T (29 ± 18.1%) and CD8 + CAR-T (71 ± 18.1%) (Itzhaki et al., 2020). Taking into consideration that on average one patient receives 3.1×10^6^ transduced viable T cells per kilogram of body weight (range, 0.2×10^6^ to 5.4×10^6^ cells per kilogram) for ALL treatment. Meanwhile, for lymphoma the recommended dose of infused CAR T-cells ranged from 0.66×10^6^/kg to 3.3×10^9^/m2 (Maude et al., 2018; Cao et al., 2019), with a normal transduction rate around 20–60% (Itzhaki et al., 2020).

### Chimeric Antigen Receptor-T Cell Immunotherapy Challenges

**FIGURE 2:**
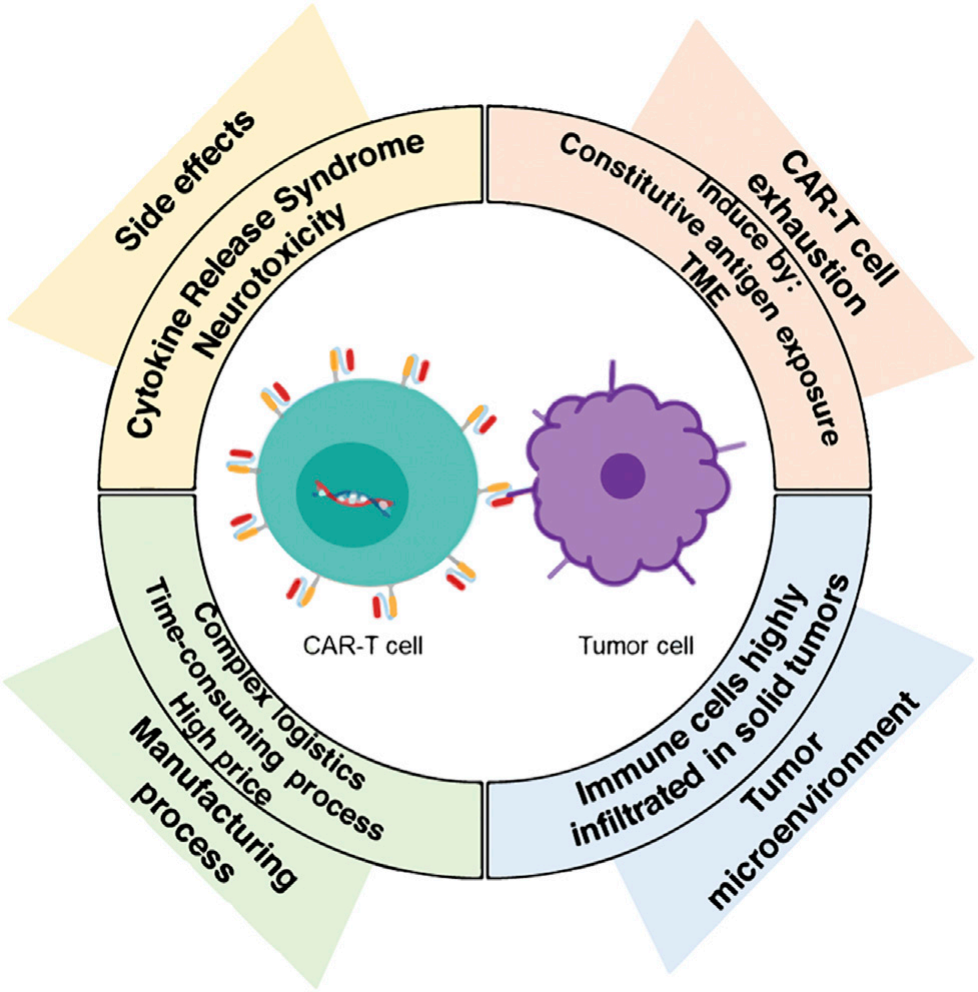
The major challenges in CAR-T immunotherapy. Nowadays, the novel CAR-T cells therapy does face some significant challenges, such as side effects, a hostile tumor microenvironment (TME), and T-cell exhaustion. In addition, the challenge of scaling-up the manufacture of clinical-grade cell therapies needs to be addressed to make them available and affordable for more patients worldwide.

CAR-T cell immunotherapy has found its success where conventional therapies fail, becoming a promising emerging technology that represents an important progress for the treatment of cancer malignancies. Furthermore, it also opens new potentials for the treatment of other diseases. However, this therapy is relatively new, and some significant challenges have been observed, mainly related to side effects, toxicity, T-cell exhaustion, and hostile tumor microenvironment (TME) (Figure 2). In addition, the manufacturing process for offering clinical-grade cell therapies at a large-scale production and their distribution are currently time-consuming and costly. Therefore, making CAR-T cell immunotherapy available for as many patients as possible becomes a greater challenge. New processing techniques, quality control mechanisms and logistic developments are required to overcome these limitations and to develop robust technology. In the next paragraphs, an analysis of such challenges is presented.

### Side Effects and Toxicity

After CAR-T cell infusion, close patient monitoring is a crucial part of the treatment protocol due to the potential lethal toxicities of this immunotherapy. Fever, inflammation, abnormal liver enzyme elevation, breath difficulty, chills, confusion, dizziness, lightheadedness, severe nausea, vomiting, and diarrhea are some of reported side effects. All patients have developed long-term B cell aplasia, which can be alleviated by administration of gamma globulins (Swiech et al., 2020; Zhao et al., 2020). There are two main categories of toxicity: Cytokine release Syndrome (CRS) and neurotoxicity (NTX) or CAR-T cell-related encephalopathy syndrome (CRES).

### Cytokine Release Syndrome

CRS or “cytokine storm” is a systemic inflammatory response, caused by the release of inflammatory cytokines, such as IL-1, IL-2, IL-6, IL-10, IL-15, interferon (IFN)-γ and tumor necrosis factor (TNF) by a large number of activated lymphocytes (B cells, T cells, and/or natural killer cells) and/or myeloid cells (macrophages, dendritic cells, and monocytes) which result in a wide range of clinical symptoms including fever, fatigue, headache, rash, joint pain and myalgia (Chavez et al., 2019; Riegler et al., 2019). CRS is the most common adverse effect that occurs within a few days after the first infusion of CAR-T cells (the CRS of any grade occurred in 85% of patients). Severe CRS cases are characterized by tachycardia, hypotension, pulmonary edema, cardiac dysfunction, high fever, hypoxia, renal impairment, hepatic failure, coagulopathy, and irreversible organ damage (Park et al., 2018). Fortunately, the effects of CRS can be attenuated by reducing the number of infused T cells and/or by the administration of anti-IL-6 receptor monoclonal antibody (tocilizumab) and steroids (Swiech et al., 2020). Nowadays, the search for different biopharmaceuticals to avoid the CRS is increasing due to COVID-19 treatment, which exhibits a similar CRS profile. This means that the next findings could be useful to improve this type of treatment.

### Neurotoxicity

NTX is another common complication of CAR-T cell immunotherapy that occurs in more than 40% of patients (Belin et al., 2020). NTX described as CAR-T cell-related Encephalopathy (CRES) by Neelapu *et al.* (2018) and, more recently described under the name of ICANS (Immune effector Cell–Associated Neurotoxicity Syndrome) (Lee et al., 2019), is less understood than CRS and usually appears within one to 3 weeks after CAR-T cell infusion, which is frequently correlated with CRS (Siegler and Kenderian, 2020). Patients show a variety of symptoms such as confusion, obtundation, tremors, delirium, word-finding difficulty, and headaches; other symptoms such as aphasia, cranial nerve abnormalities and seizures have also been reported as part of NTX effect (Neill et al., 2020). Nowadays, neurotoxicity management is treated by administering tocilizumab: an IL-6 receptor antibody; systemic corticosteroids; and dexamethasone (Gust et al., 2018).

The prompt management of toxicities is essential to minimize the mortality associated with this immunotherapy, for that, researchers have developed different safety strategies for overcoming and prevent the CAR-T cells toxicities such as the design of new CARs generations (Andrea et al., 2020; Rafiq et al., 2020). Toxicity management has become a critical step in the success of CAR-T cell immunotherapy. In fact, Tmunity Therapeutics developed a dual PSMA-specific, TGFβ-resistant CAR-modified autologous T cells (CAR-T-PSMA-TGFβRDN) for prostate cancer with the objective to expand the use of CAR-T from hematological malignancies to solid tumors. This clinical trial (NCT04227275) had a setback that led to its winding down after two patients died from a type of ICANS. Nevertheless, the study helped to identify the potential barriers of CAR-T therapies against solid tumors, which results in a better understanding of the adverse effects mechanisms that will aid to design safer candidates.

### Chimeric Antigen Receptor-T Cell Exhaustion

CAR-T cells have raised enormous interest in cancer immunotherapy due to the high rates of complete remission. Nevertheless, a large fraction of patients that achieve remission have displayed disease relapse within a few years (Maude et al., 2018; Park et al., 2018), the relapse rate varies from 21 to 45% in B-ALL and increases with the follow-up time (Cheng et al., 2019). The treatment failure could be partially explained by CAR-T cells exhaustion induced by tumor microenvironment (TME) created by solid tumors. Similar phenomena appear to extend in hematological malignancies including chronic lymphocytic leukemia (CLL), acute myeloid leukemia (AML), and diffuse large B cell lymphoma (DLBCL), besides to an excessive antigen exposure (Poorebrahim et al., 2020; Shen et al., 2020). The complex and heterogeneous TME affects the activation and function of the infiltrated effector T cells and thus impairs the persistence, proliferation, and potential of the T cells rendering them exhausted (Tahmasebi et al., 2019). CAR-T cells exhaustion refers to a state of dysfunction characterized by physical deletion of antigen-specific T cells due to persisting antigen stimulation, the costimulatory domain of CAR structure and increased expression of inhibitory receptors, usually induced by chronic stimulation, such as cancer (Wherry, 2011; Cheng et al., 2019). *In vitro* CAR-T cell studies have shown that during CAR-T cell exhausting process the loss of functionality against tumors is mainly triggered by the upregulated expression of inhibitory receptors such as PD-1, Lag3, Tim3 TIGIT, and the inhibition of PI3K/AKT pathway through CTLA-4. Nonetheless, cytokines play an important role in T cell activity or exhaustion, for instance, CAR-T cells decrease the ability of interleukin 2 (IL-2), Tumor necrosis factor α TNF- α, and interferon–γ (IFN-γ) among other cytokines (Tang et al., 2021); Interleukin-5 (IL-5) increased proliferative capacity, decreased apoptotic state through IL-5 mediated reduction of mTORC1 activity (Alizadeh et al., 2019).

Other factors such as transcriptional factors, metabolism, and epigenetic modification also play an important role in CAR-T cell exhaustion development (Shen et al., 2020). This dysfunctional phenotype is associated with hallmarked loss of the CAR-T cell’s capacity of expansion and persistence. That situation compromises the patient’s clinical remission (Fry et al., 2018). The reason why CAR-T cells lose *in vivo* persistence and potency remains unknown (Balkhi, 2020), that is why the CAR-T cells exhaustion is a pivotal hurdle for successful CAR-T cell therapies. A possible approach to delay the exhaustion is the engineering of exhaustion-resistant CAR-T cells. Recent reports indicate that the discovery of certain transcription factors like TOX (Seo et al., 2019) and NR4A (Cheng et al., 2019), and the deletion or overexpression of AP-1 family transcription factor c-Jun (Lynn et al., 2019) increases CAR-T cell resistance to exhaustion. Some studies have shown that CAR-T cells equipped with cytokines can enhance their own lifespan and expansion, promoting their own growth and proliferation (Avanzi et al., 2018; Zhao et al., 2020). That is the reason for the TRUCKs development by genetically adding an inducible cytokine-producing cassette (e.g., IL- 12) (Zhao et al., 2020). Lately, deleting PD-1 by CAR-T cells engineering (short hairpin RNAs or CRISPR/Cas9), blocking antibodies using PD-1 and blocking the IFN-γ signal have been used to increase the CAR-T therapy efficacy avoiding exhaustion (Cheng et al., 2019; Rafiq et al., 2020). In this case, the discovery of the cellular mechanisms involved during cell proliferation and the maintenance of cell viability are a hindrance to the functioning of this therapy.

### Tumor Microenvironment

The success of CAR T-cell immunotherapy has not yet been achieved in solid tumors. A possible reason is that the immunosuppressive nature of the tumor microenvironment (TME) affects the efficacy of adoptive immunotherapy. Solid tumors are highly infiltrated with stromal cells like cancer-associated fibroblasts (CAFs) and suppressive immune cells, including myeloid-derived suppressor cells (MDSCs), tumor-associated macrophages (TAMs), tumor-associated neutrophils (TANs), mast cells, and regulatory T cells (Tregs) which contribute to the establishment of a hostile and immunosuppressive TME capable of interfering the efficacy of CAR-T cell therapy (Martinez and Moon, 2019; Rodriguez-Garcia et al., 2020). Strategies to overcome TME effect, include enabling T cells to resist tumor suppression in TME, such as transgene expression of dominant-negative receptors or signal converters, which can transform suppressive signals into stimulating signals (Tang et al., 2021). Another opportunity to overcome the persistence and exhaustion of CAR-T cells is improving the trafficking delivery of the drug to the tumor site. For CAR-T cells, local injection is a popular investigation among trials. This could be done in an anatomical cavity (pleura, peritoneum), via a device placed surgically (for CNS tumors) or via intra-arterial delivery (such as hepatic artery catheterization), or by direct intratumoral injection (Edeline et al., 2021).

### Genetic Alterations

Several reports have highlighted a lack of efficacy and relapses in patients treated with CAR-T cells (Sotillo et al., 2015; Fischer et al., 2017). Few reports have tackled the identification of genomic modifications post CAR-T cell therapy due to vector integration (Nobles et al., 2020). Orlando et al. (2018) incorporated whole-exome DNA-seq and RNA-seq to investigate the extent to which CD19 mutations are the main mechanism causing relapse. They discovered *de novo* CD19 genetic alterations in exons 2–5 in 12 of 12 samples from patients, and loss-of-heterozygosity in 8 of 9 patients, concluding that homozygous mutations in CD19 are the main reason for acquired resistance to CAR-T cell therapy. Asnani et al. (2020) reported similar findings; they described skipping of exon 2 and exons 5-6 in patients with relapsed leukemia after CAR-Tc therapy. Exon 2 is essential for the integrity of CAR-T 19 epitope while exons 5-6 are responsible for the CD19 transmembrane domain (Bagashev et al., 2018). CAR-T cells have been shown to improve the efficacy of immunotherapy; however, further research is needed to explore the impact of genome analysis to identify the prognosis once CAR-T cell transfusion is performed.

### Manufacturing Process

**FIGURE 3:**
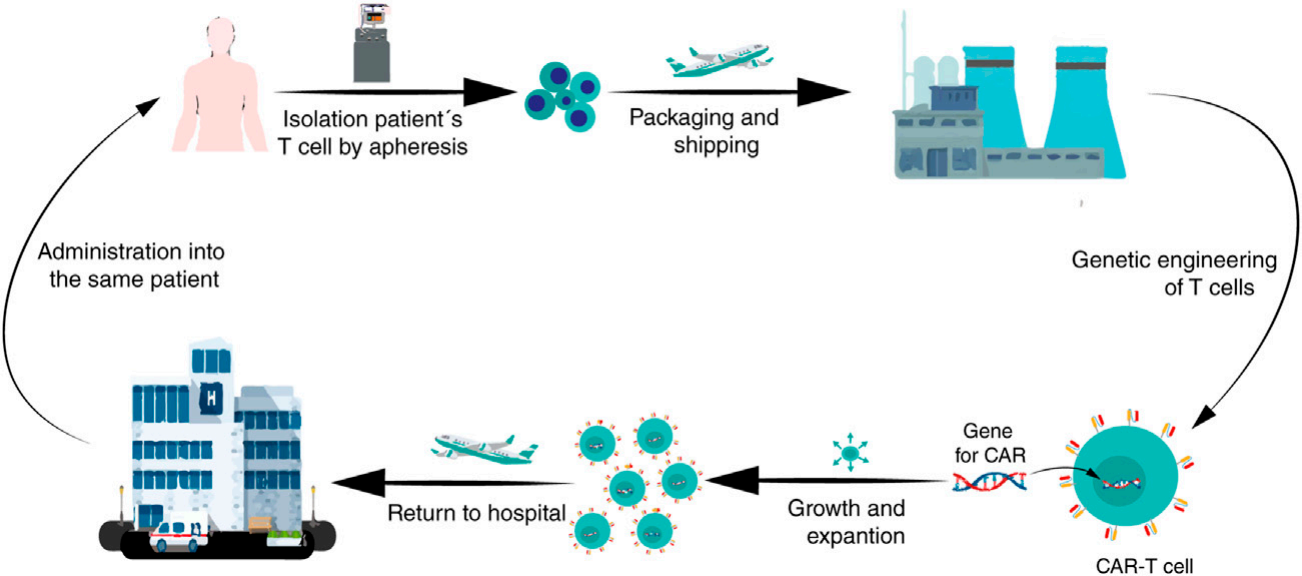
A brief diagram of the CAR-T cells manufacturing process. After enough leukocytes have been harvested from a patient’s blood via leukapheresis, T cells are shipped to a biopharmaceutical company where they are genetically engineered to express a CAR on the T cell surface. Then, the CAR-T cells are amplified *in vitro*; after that, the CAR-T cells are returned to the hospital for administration into the patient.

The traditional technology to manufacture CAR-T cells consists in: 1) to isolate patient’s T-cells (autologous) by apheresis; 2) to ship recovered cells to a central manufacturing site (bio-pharmaceutical companies) where 3) they are genetically engineered to express a CAR after which 4) they are expanded in the laboratory; then, 5) the CAR-T cells are returned to a hospital to be 6) infused into the patient for tumor eradication (Figure 3) (Roddie et al., 2019). The logistics involved in this traditional manufacturing and treatment with autologous CAR-T cells adds complexity for clinicians and patients because the period in between, referred to as *“vein-to-vein time”*, ranges between three and 4 weeks in developed countries. This period can be daunting for the patients awaiting treatment and renders the CAR-T treatment complicated for patients with rapidly progressing diseases (Aftab et al., 2020). Nowadays, this therapy presents these main manufacturing challenges and the picture in developed and underdeveloped countries is very different. Some of such challenges include:

### Packaging, Shipping, and Storage of Chimeric Antigen Receptor-T Cells

The clinical manufacture of CAR-T cells is currently a complex process that involves several steps across different geographic locations with multi-technologies and logistics. Any error in timing, transportation methods, cold chain, or storage, would result in cell damage directly impacting therapy efficiency, hence each one of the steps requires careful management, precise sample tracking and adequate preservation technologies to either freeze or cryopreserve patient samples (Papathanasiou et al., 2020). Different shipments with dissimilar temperatures are required throughout the CAR-T cell manufacturing process, therefore, suitable cryopreservation during production is mandatory for quality control tests (Stock et al., 2019).

### Current Good Manufacturing Practices

CAR-T cells have a complex preparation process and the cGMP´s are a critical and a bottleneck in CAR-T cell production (Wang and Rivière, 2016). The aim of cGMP´s is to provide a framework to ensure high-quality production in well-controlled facilities and equipment by well-trained and regularly trained staff. Likewise, it provides strict documentation processes that covers all operation aspects to demonstrate a continuous and adequate compliance (Li et al., 2019). The CAR-T cells manufactured under GMP regulations should provide a system in which the cells will be prepared under controlled, auditable, reproducible conditions that result in providing adequate evidence of identity, safety, purity, and potency (Gee, 2018). The manufacturing protocols must improve reproducibility, cost-effectiveness, and scalability that will enable a broad application of CAR-T cell therapies (Fernández et al., 2019). According to International Organization for Standardization (ISO)5 CAR-T cell manufacturing requires GMP facilities as cell processing clean rooms, that must be equipped with 1) Facilities systems (e.g., air-handlers, 24/7 alarm monitoring systems); 2) Environmental monitoring equipment (e.g., viable and nonviable particle counters); 3) Manufacturing process equipment (e.g., cell washers, bioreactors); and 4) Analytical equipment (e.g., automatic cell counters, flow cytometers) (Wang and Rivière, 2016). To optimize the GMP facilities the use of automatized manufacturing processes as CliniMACS or Cocoon is feasible to achieve reproducible processes in a closed system. Another key to maintaining a compliant GMP manufacturing environment is the highly skilled staff with extensive knowledge of GMP manufacturing, quality control, and quality assurance (Wang and Rivière, 2016). One complete review of the requirements for CTL019 is presented by Levine et al. (2017), who explain the complete manufacturing process and the challenges involved in each stage.

### Production of Lentiviral Vectors

The approval of LV gene therapy products (*e.g*., Kymriah/Novartis) increased their demand and has created the need to improve their large-scale manufacture to clinical-grade LV (Lester et al., 2018). Nevertheless, the LVs production has challenges, such as their inherent cytotoxicity, low stability, and the dependency on transient transfection impact, both upstream and downstream processes that are reflected in low yielding and cost-ineffective compared to another viral vector (Ferreira et al., 2021). The level of GMP compliance required for the manufacture of LV is a combination of diverse factors including regulatory expertise, compliant facilities, validated and calibrated equipment, starting materials of the highest quality, trained production personnel, scientifically robust production processes, and quality by design approach (Levine et al., 2017; Dasgupta, et al., 2020). In addition, part of the commercialization success of this gene therapy product is the establishment, standardization, and implementation of stable cell lines to produce LVs facilitating GMP-compliant processes, providing an easier scale-up, reproducibility, biosafety, and cost-effectiveness (Ferreira et al., 2021).

### Staff and Training

Considering the complex nature of the therapy and its associated high-risk side effects, access to CAR-T cells is highly regulated, making them available only at certified centers and administered by trained staff. All staff involved in CAR-T cell manufacturing (from T cell collection to the manufacturer and back to the clinical unit) require a broad training with satisfactory levels of competency. Such abilities manage complications that could arise during the process hence being able to deliver the product (Yakoub-Agha et al., 2020). Nowadays, there are only a few prepared professionals in this field, however, multidisciplinary collaboration is needed to create greater knowledge in this area. Academic participation is an important fact because the new personnel need to acquire different abilities to manufacture CAR-T cells.

### Quality Control

**FIGURE 4:**
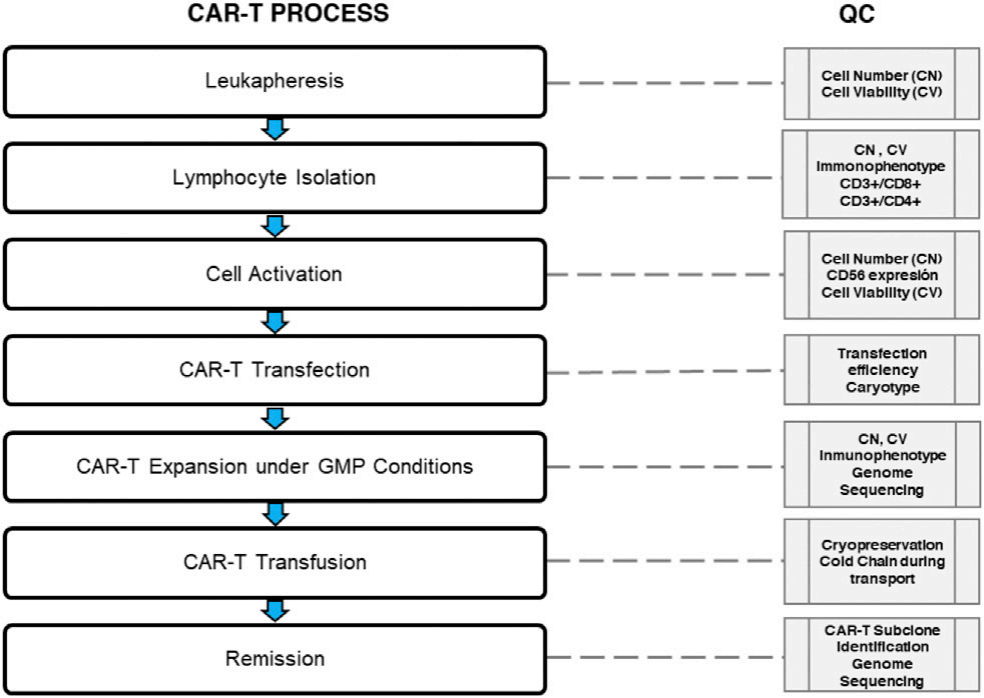
Quality control during CAR-T cell therapy. The median of total nucleated cells (TNC) is 98 × 10^8^ (range 9 - 341 × 10^8^) with a viability analysis of 98% for quality control (QC) in the leukapheresis products. However, the QC could be set in 10× 10^8^ CD3^+^ T cells with a viability analysis of 98% (99.6–100%) in a median volume of 237 ml (136–310 ml) for CD3^+^ T cell isolation (Data based in the report of Korell et al., 2020). The normal transduction rate is around 20–60% using lentiviral vectors. A 2:1 ratio immunophenotype of CD4+: CAR-T (29 ± 18.1%) and CD8+: CAR-T (71 ± 18.1%) is recommended for clonal CAR-T cell expansion under GMP conditions. Clonal expansion must reach 3.1×10^6^ transduced viable T cells per kilogram of body weight (range, 0.2×10^6^ to 5.4×10^6^ cells per kilogram); this CAR-T cells product is ready for cryopreservation. Cold chain must be controlled during transportation using liquid nitrogen. Prior infusion lymphodepleting chemotherapy with fludarabine and cyclophosphamide it is used. Finally, rate a CAR-T subclone identification followed by genome sequency should be evaluated for measure the molecular remission.

As a living ‘‘drug,” a CAR-T cell has a complex preparation process and requires “whole-process quality control”. During the production, well-controlled cold chain transport and storage play an important role in ensuring the cell products’ quality and preventing the bacteria and *mycoplasma* contamination (Li et al., 2019). Current requirements in the CAR-T cell´s quality control include the inspection of *ex vivo* transduced T-cell for the replicative virus and of the production materials. Furthermore, it should include a release test of the finished products to confirm identity, purity, safety, and potency, considering CAR-T cell´s characteristics as biological products, cell products, and gene therapy products (Figure 4) (Eyles et al., 2019; Li et al., 2019). In addition, the validation of the production process is essential for CAR-T cells to be considered a final product. Moreover, stability studies are needed to verify the storage conditions and their shelf life. Besides, the production of CAR-T cells needs more in-depth studies to evaluate T cells’ quality in both relapsed and regression patients. These studies should provide data about the distribution of lymphocyte populations. This information can guarantee efficient cell transduction and propagation ensuring the CAR-T cells’ quality, and subsequently it would be reflected in the decrease of CAR-T cells exhaustion in patients. To sum up, quality control is of ultimate importance for the success of CAR-T therapy (Dai et al., 2019).

### Scaling Up

CAR-T cells manufacturing should be scalable (that means, to have many single bioreactors for each patient) to have a broader scope to benefit patients without sacrificing the quality and reproducibility of the products (Dai et al., 2019). Personalized therapies such as autologous cell-therapies require an “intensive scale-up”, that is related to the fact of having many bioreactors to amplify the CAR-T cells for each patient, instead of increasing the volume as in common biopharmaceuticals. Besides, it depends upon the ability to accommodate multiple independent productions in parallel (Wang and Rivière, 2016).

### Limited Opportunity for Redosing

CAR-T cells manufacturing does not allow volumetric scale-up, consequently, cells must be prepared as a single batch limiting the quantity of available product. Under this scenario, patients may not have the opportunity to receive a new infusion of their CAR-T cells quickly and easily if needed (Aftab et al., 2020; Papathanasiou et al., 2020).

### Time Manufacturing

CAR-T cells can take up to 4 weeks to manufacture, during this window of time, the patients are extremely vulnerable with risk of disease progression and mortality while waiting for infusion (Rafiq et al., 2020). Nevertheless, the use of novel strategies could help to overcome such a limitation, but it is necessary to consider the time required for transporting to prepare the adequate conditions for the patient. This takes around 6 weeks for the treatment to be applied at best.

### Pricing and Patient Access

Pricing and patient access is the most important limitation to overcome to disclose the use of CAR-T cells all over the world. The current model of CAR-T cell manufacturing is highly centralized: firstly, the patient’s T cells must be transported (often internationally) to the manufacturing facility, secondly, the CAR-T product is returned to the patient for infusion (Hay and Cheung, 2019). The process is complex in each step, resulting in highly costly therapy that ranges between $373,000 and $475,000 USD per treatment (hospital expenses associated with the therapy are not considered in such average costs) that neither patients nor the health-care system can afford. This prohibitive cost and the reimbursement gap limit the patient’s access and remain unsustainable in socio-economically underdeveloped countries, restricting even more the widespread use of CAR-T cell therapies. Until CAR-T cell therapy becomes affordable, its potential will remain untapped (Yadav et al., 2020).

### Regulatory Requirements

Another important bottleneck in cellular products is regulation. The CAR-T cells are globally considered within Advanced Therapy Medicinal Products (ATMP) and these products require licensure (Marks 2019). Regulatory agencies are highly related to standard therapies, but cellular products have special requirements. US or EU regulatory authorities are working to define optimal guidelines that can globally harmonize the requirements for clinical manufacturing of ATMP (Kaiser et al., 2015). Meanwhile underdeveloped countries face a greater challenge because CAR-T therapeutic use in clinics is hugely restricted, resulting in a poor understanding of the regulatory requirements by the authorities.

### Strategies to Increase the Use of Chimeric Antigen Receptor-T Cells Technology. Discovery of New Biomarkers

Biomarkers are of main importance for cancer clinical management, as they can be used to define the criteria to identify the patients suitable for CAR-T therapy, prognosis, prediction of response to treatment and monitoring disease progression. Developing cancer immunotherapy has therapeutic implications and its success varies depending on the type of cancers (Schumacher et al., 2015). On the other hand, CAR-T cell immunotherapy has achieved promising results mainly in patients with hematologic malignancies. On the other side, this type of therapy has more obstacles attacking solid tumors, including immunosuppressive tumor microenvironment (TME), lack of permeation of the CAR-T cells into tumors, limited transport, and specific tumor antigen (Ling, 2020). Despite the significant advances, clinical improvement after CAR-T therapy has not been observed for all the patients who have undergone this immunotherapy. Such differences could rely on molecular response; hence it is necessary to fully understand remission and relapse in CAR-T therapy through biomarkers. Consequently, it is essential to identify the clinical features in the form of biomarkers that drive response, resistance, and adverse effects because of immunotherapies (Townsend et al., 2018). Thus, for the respective CAR-T cell immunotherapy development and success it is essential to broaden the range of cancers that respond to such a kind of immunotherapy with the discovery of new biomarkers. The identification of new biomarkers improves clinical practices for early recognition and minimization of adverse effects while preserving the antitumor activity of the CAR-T cells (Mirzaei et al., 2018), it opens the possibility to target other types of tumors and leads to the development of new strategies for numerous types of cancer treatments, positioning CAR-T cells as a therapeutic option in cancer medicine (Ponterio et al., 2020). The first biomarker for CAR-T - therapy was CD19, a B cell surface protein expressed mainly on malignant B cells. Currently, scientific research is underway to find out biomarkers according to the stage of immunotherapy as follows: biomarkers to define patients baseline status; biomarkers for CAR-T-cell function, CAR-T cell exhaustion, CAR-T cell toxicity and biomarkers for cancer prognosis, response, and relapse (Mirzaei et al., 2018). As for baseline biomarkers, cytokines of the immune system such as IL2, IL-5, IL-7, TNF-a, among others; lactate de Lactate dehydrogenase (LDH) and CD9 cells, are widely used. Meanwhile, for CAR-T cell function, the following biomarkers have been proposed: CD45RA, CD45RO, CD62L, CCR7, CD27, CD28 (differentiation markers), CD25, CD127, CD57, and CD137 (activation markers) (Wang et al., 2019; Hong et al., 2021). Currently, there are no proven biomarkers that can be used to assess CAR-T cell exhaustion after infusion in patients. However, indirect parameters could be helpful for this purpose; for example, a sustained gain in B-cell recovery within 3 months after CART-therapy and the detection of LAG3+ cells could be considered due to CAR-T cell exhaustion. As well as high levels of expression of PD-1, LAG-3, TIM-3, and their receptors are associated with T cell exhaustion and poor response to CD19 CAR-T therapy (Hong et al., 2021). Recently a model of T cell exhaustion assessment through flow cytometry has been proposed based upon PD-1 pathway blockade response, identifying three main stages: exhausted progenitors, intermediated exhausted, and terminally exhausted T cells (Beltra et al., 2020). Relapse: IL-6, Protein C reactive, TNF-a, serum calcium, serum phosphate, uric acid (Zhang et al., 2016). In Table 3, a summary of novel target antigens selection for CAR-T cells in hematologic malignancies and solid tumors under clinical trials is presented. Despite significant advances in CAR-T therapy, it is essential to continue exploring different cancer cell-type-specific biomarkers to develop more specific therapies.

### Allogeneic Chimeric Antigen Receptor-T Cells

Currently, most CAR-T cell immunotherapy is generated using autologous T cells. This represents several disadvantages at different levels. For example, autologous cell therapy is performed for individual patients, the production can be long-time consuming and complicated, resulting in the increase of costs and it can limit the broader application of this therapy. In addition, the challenges due to the use of patient-derived T cells for CAR therapy include weaker proliferation, limited expansion, and low persistence of CAR-T cells. In some cases, CAR-T cell efficacy, most likely due to poor autologous immune cell strength in cancer patients caused by aggressive treatments (Caldwell et al., 2021). One opportunity for improving some manufacturing-related issues is the use of allogeneic CAR-T cells for reducing time delays on autologous cell production. Generating universal CAR-T cells from allogeneic healthy donors could set the stage for overcoming high costs and high time production, making them easily available and with higher quality. Such a fact is very important for patients with aggressive cancers that require urgent therapy (Caldwell et al., 2021). This strategy would broaden the number of patients that could receive this immunotherapy to make CAR-T cell therapy an off-the-shelf treatment at an affordable cost, that be readily available and would increase quality properties in T cells (Cheng et al., 2019). Another advantage is that universal CAR-T cells could provide the possibility of manufacturing clinical-grade CAR-T products against various cancers “on-demand” (Balkhi, 2020). However, it is important to also consider the up-coming challenges and disadvantages for using universal CAR-T cells. For example, immunologic mismatch between donor and recipient, if the administered allogeneic T cells attack healthy recipient tissues, may cause life-threatening graft-versus-host disease (GVHD), and if the recipient’s immune system recognizes and reacts against the allogeneic T cells, these may be rapidly eliminated by the host immune system limiting the expected therapeutic effect (Depil et al., 2020). A possible solution is a genetic elimination or disruption of the TCR gene and/or HLA class I loci on the donor to eliminate GVHD (Cheng et al., 2019), also, the use of allogeneic anti-CD19 CAR with deleted α-TCR chain and CD52 gene loci has demonstrated effectiveness to eliminate GVHD (Cheng et al., 2019; Balkhi, 2020).

Different allogeneic strategies have appeared to improve immunotherapy challenges such as the use of induced pluripotent stem cells (iPSC). iPSCs offer the opportunity to be unlimitedly cultured *in vitro* and be successfully differentiated towards the lymphoid lineage as to NK lymphocytes that are innate lymphocytes that kill malignant cells in an HLA-independent manner (Kennedy et al., 2012). The use of iPSCs provides unlimited ‘‘off-the-shelf’’ NK cells offering doses without limitations, standardized and cost-effective products (Zeng et al., 2017). iPSCs can generate immune effectors with the capacity of easily being genetically modified to augment their applicability, potency, and persistence (Nianias and Themeli, 2019). The possibility of effortlessly generating NK cells from iPSCs (iPSC-derived NK cells) opens the great option to use the CAR constructions as an alternative to T cells, to produce CAR-iPSCNK cells with rapid responses against malignant cells without causing GVHDs (Euchner et al., 2021). Compared to CAR-T cells, CAR-NK cells offer some more advantages, such as a lack or a decrease in cytokine release syndrome and neurotoxicity in an autologous setting, another point in its favor is the multiple mechanisms for activating cytotoxic activity (NKG2D, KIR´s, CD16, NKp30, NKp44, NKp46), still maintaining its infiltration capacity into solid tumors and into the resistant tumor microenvironment (Xie et al., 2020). CAR-NK preclinical studies showed effectiveness against hematologic malignancy targets (CD19 and CD20), as well as solid tumor targets demonstrating their potential to be an allogeneic therapeutic (Caldwell et al., 2021). Besides CAR-iPSCNK strategy, virus-specific T cells, memory T cells, genetically modified αβ T cells, and *γδ* T cells are other effective ways to tackle allogeneic CAR immunotherapy. All these strategies have in common their effectiveness to attack malignant cells, and the possibility to use CAR construction to improve toxicity effects, costs and efficiency of CAR-T manufacturing, and the persistence of reducing GVHD risk (Martinez and Moon, 2019; Yazdanifar et al., 2020).

### Other Strategies to Improve Chimeric Antigen Receptor-T Cells Outcomes

Dual or tandem CARs, consist of the coexpression of two separate CARs in each T cell, which recognize two different antigens rather than one. Some dual CARs have entered clinical trials in hematological malignancies targeting CD19/CD20, and CD19/CD22 (Zah et al., 2016; NCT03241940) and solid tumors. CAR specific for HER2/MUC1 had promising *in vitro* results in a breast cancer model. It is important to mention that dual-target CAR specific HER2/IL13Ra2 showed greater success than single-target CARs in a xenograft glioma model (Martinez and Moon, 2019). Dual CAR is a promising method to address antigen heterogeneity and to prevent the relapse caused by antigen escape (Huang et al., 2020). Synthetic Notch (synNotch) receptors have been applied in CAR-T cells to improve safety. SynNotch receptors recognize one specific tumor antigen and then transcriptional activation domains are released, promoting the local expression of a CAR (Han et al., 2019). Moreover, synNotch-regulated CAR expression prevents constitutive signaling and exhaustion, maintaining a higher fraction of the T cells in a naïve/stem cell memory state (Choe et al., 2021). Synthetic Notch (synNotch) receptors have been applied in CAR-T cells to improve safety. SynNotch receptors recognized one specific tumor antigen and then transcriptional activation domains were released, promoting the local expression of a CAR (Han et al., 2021). Moreover, synNotch-regulated CAR expression prevents constitutive signaling and exhaustion, maintaining a higher fraction of the T cells in a naïve/stem cell memory state (Choe et al., 2021). Inhibitory chimeric antigen receptor (iCAR), which incorporates inhibitor receptors, such as PD-1 and CTLA-4, play a crucial role in attenuating or terminating T cells response, therefore, they are considered a safety strategy (Yu et al., 2019). iCAR shows antigen-specific suppression of T cell cytokine secretion, cytotoxicity, and proliferation of rapid and selective kills of target cells that only express one antigen, whereas off-target cells, that co-expresses another inhibitory ligand recognized by iCAR, are protected from attack. This allows T cells to distinguish target cells from the off-target cells (Han et al., 2019). With this said, it is important to highlight the following aspects for a successful clinical application: the iCARs’ affinity and expression level, the appropriate selection of target antigen and the optimization of the CAR/iCAR ratio (Gargett and Brown, 2014). Currently, Jan and *cols.,* have developed ON and OFF switches for CAR-T cells using lenalidomide, a clinically approved drug, which facilitates the proteasome degradation of several target proteins (Jan et al., 2021). In addition, to efficiently treat solid tumor, innovative combinations strategies such as vaccines, biomaterials, and oncolytic virus are good prospects, because they allow either to directly enhance T cells’ function or to recruit endogenous immune cells as well as remodeling the TME (Huang et al., 2020).

### Fully Automated Manufacturing Process

Worldwide, the number of patients who require CAR-T cell immunotherapy quickly increases. That is the reason why the standardization of the manufacturing process (following GMP’s), the quality control and expanding the production to hospitals making the treatment accessible for a larger number of patients, are highly needed. The industry has developed automated and closed cell manufacturing platforms to adapt to this scenario. Examples of this effort are the automatized platforms Cocoon® (Lonza) and CliniMACS Prodigy (Miltenyi Biotec), both allow reproducible and rapid production of cells, and every step is strictly documented. Cocoon® Platform streamlines patient-scale cell therapy manufacturing making them more efficient, reliable, and flexible. These features speed up CAR-T manufacturing, cut costs and decrease manufacturing failure rates that may be associated with more hands-on methods. In 2020 Lonza and Sheba Medical Center announced the first patient has been treated at Sheba Medical Center with CD19 CAR-T cell immunotherapy manufactured using Lonza’s Cocoon Platform. Another example for automated and supervised manufacturing is CliniMACS, which allows T cell activation, transduction, amplification, and final harvesting of CAR-T cells, with high transduction frequencies and high cell numbers in the same device. This method fully controls the multi-step process through a flexible programming suite (Lock et al., 2017; Köhl et al., 2018).

### Network Collaboration

Unfortunately, most of the patients cannot afford CAR-T therapy due to the high costs and the lack of facilities around the world to produce them. The efforts focused on quality production following the GMP’s had been successful to a certain extent, but the road is still long to bring this technology to a massive application.

To achieve this, it is necessary to generate a collaborative network between various stakeholders such as the academia, the industry, and the hospitals, for the establishment of adequate and robust legislation for this type of product in each country where such a technology is applied. Thus, universities should provide knowledge and innovation to future professionals for them to do research on new biomarkers discoveries, on the development of stable cell lines, and on developing and validating orthogonal analysis methodologies for the product quality and for them to learn about different production systems. The industry applying GMP’s, quality control and automation processes can support the establishment of more standardized and reliable products that have a high quality, and it can also develop on-site production units that allow lower transport and storage costs. Hospitals must have adequate facilities for the management of this technology, with trained personnel in the management of production units *in situ* (if applicable), as well as medical personnel and health professionals for the effective treatment of patients. Encompassing all these activities there must be robust and specific legislation in this area that guarantees the quality of this type of advanced therapy. Additionally, regulation is an essential part of such a process. Nowadays, there is sparse information about regulatory guidelines in different countries, the EMA and FDA guidelines (Iglesias-López et al., 2019) have established the baseline of such requirements for these technologies but the behavior and results on different phases of clinical trials will address novel considerations. One challenge but at the same time one opportunity will be to establish local regulation of these technologies in every country that needs to be approved by competent authorities for smooth implementation of CAR-T cell clinical programs. All this collaborative effort is the key to allow worldwide access to CAR-T cell therapy to a larger number of individuals with cancer.

Finally, the idea of promoting the use of CAR-T cells technology in a greater number of countries rests on its potential application in various diseases in both adults and children. To our knowledge, the application of CAR-T cells technology has been mainly used in developed countries (Elahi et al., 2018), revealing a large gap in the rest of the world. In fact, the only clinical study conducted in Latin America was made in Brazil (Cunha Oct 16, 2019) in a 63-year-old man that was affected by BLBCL. Therefore, it is important to consider that for this technology to be enforceable in developing countries various policies must be created and implemented. On the one hand, technology transfer agreements can be established among centers with experience in the subject and treatment centers in developing countries, however, this continues to foster scientific-technological dependence. On the other hand, developing countries should work on integrating their own elements (academy-industry-hospital and government), generating treatments of this type but geared to the specific needs of its population and adapting them to the available resources. However, it is important to highlight that in this case, government support is essential to start setting up treatment centers for advanced therapies in hospitals and health institutions. In addition, international support from non-profit institutions in terms of financial resources and support for the training of professionals is also necessary.

## Discussion

Current and widely used methods for cancer treatment consist of surgery, chemotherapy, radiation therapy and immunotherapy (mainly monoclonal antibodies). Despite the health care system efforts, with the increasing incidence of cancer, its clinical management continues to be a major challenge. Recently, remarkable progress has been achieved towards adoptive immunotherapies through clinical application and consistent evolution of ACT strategies. Such immunotherapies, in combination with conventional methods have proven major efficiency for cancer treatment. In the last few years, CAR-T cell therapy, a type of ACT, has demonstrated potential in clinical trials, leading to complete and durable responses in patients with late-stage and treatment-refractory disease. In fact, there are currently 5 FDA-approved products on the market, which have been tested in a few hundred patients during clinical trials in developed countries around the world. Furthermore, the first one was approved for commercial use in 2017 but the cost of such a treatment is substantially high (from $ 375,000.00 to $475,000.00 without considering operation and indirect costs) so, considering its potential application in the treatment of different types of diseases there is a need to improve the technology to make it more accessible to the world’s population.

From a pharmaceutical point of view, CAR-T cells are considered as advanced therapy products or biopharmaceuticals (depending on the country) whose quality must be demonstrated through their identity, safety, and efficacy. This novel technology is the set of synergy between three main elements: the targets (antigens against the CAR will be directed and that, ideally, are required to be only expressed in malignant cells); chimeric receptors (whose sophistication allow to bind to said antigen and that are designed to provide better selectivity towards tumor cells, and properly, T cells (containing CD3 +/CD4 + and CD3 +/CD8 +, in an adequate proportion) that are genetically modified to express the CAR and guarantee the success of such therapy.

Each of the previous three elements presents significant challenges. As discussed in this work, the search for biomarkers is an exhaustive task aimed at identifying target molecules that are specific to a certain type of disease, which will help improve efficacy and reduce adverse effects. Likewise, the improvement in the development of CARs to promote an effective union with the receptor and the generation of an effective immune response against the disease and improve activation, proliferation, and survival is the subject of study. Furthermore, many efforts are directed at the selection of the T cells used in this technology, such cells must comply with adequate phenotypic characteristics; being genetically stable; being able to transduce within a high percentage (also putting on the table the need of vector optimization for this purpose); being expanded to reach the required levels for the therapy; being enough stable to avoid the cell exhaustion; and, accomplishing all the requirements of a cell therapy product at the level of identity, purity and efficacy. Furthermore, it is necessary to focus on the reduction of adverse effects related to CRS and NTX and in the application of this technology in solid tumors with the idea of increasing the safety and efficacy of this therapy, among other points of improvement.

Alternatively, the need to generate lower-cost production systems, with robust production in each batch (patient-specific), but that meet the necessary quality requirements to be applied to the patient is also a constant challenge. Currently, the use of automated systems applicable *in situ* seems a promising alternative. These processes must be in complete harmony with the requirements established by international and regional health authorities.

Finally, considering all the aspects of this therapy, the multidisciplinary participation of health care professionals in various stages is required. Through intensive collaborations between academia, industry, hospitals, and government, both at the international and regional level, it will be possible to achieve a broader accessibility to this technology to a larger number of patients. This situation should be able to overlap in the case of developing countries, who must seek different strategies by applying their own generated knowledge, making an efficient use of the existing infrastructure, and seeking collaborations at various levels to obtain the necessary information and resources. The implementation of these strategies is likely to increase the number of treatments all over the world.
